# Vagus nerve stimulation modulates LPS-induced epileptogenicity: the role of inflammation suppression

**DOI:** 10.21203/rs.3.rs-8896240/v1

**Published:** 2026-04-20

**Authors:** Georgia Lawlor, Sadid Khan, Thalis Asimakopoulos, Irika Sinha, Jonathan Ling, Pedro Irazoqui, Athanasios Alexandris, Vassilis Koliatsos

**Affiliations:** The Johns Hopkins University; The Johns Hopkins University; The Johns Hopkins University

**Keywords:** neuromodulation, vagus nerve stimulation, inflammation, seizures

## Abstract

Sustained systemic inflammation causes neuroinflammation and increases seizure risk, yet mechanisms linking inflammation and epileptogenicity remain poorly understood. Vagus nerve stimulation (VNS) suppresses systemic cytokines and modulates microglial activity after acute inflammatory challenges, but it is unknown whether these effects persist with sustained inflammation. Here we employed daily VNS in a rat model of endotoxemia induced by five daily lipopolysaccharide (LPS) injections. Rats received VNS from an implanted, wirelessly powered neurostimulator. Seizure susceptibility was assessed with pentylenetetrazol infusion, and peripheral and central inflammation were evaluated with serum cytokines, microglial cytology, and transcriptomics. Our findings show that sustained LPS exposure lowers seizure thresholds and induces strong systemic and central inflammatory responses. Our VNS regimen suppressed epileptogenicity, elevated serum IL-10, and shifted splenocyte gene signatures toward quiescence but had only subtle, region- and sex-specific effects on microglia and central inflammatory markers. These results suggest that VNS can suppress sustained systemic inflammation and mitigate inflammation-associated epileptogenicity, although its anti-epileptic effects may also involve non-neuroinflammatory mechanisms. A caveat is that sustained LPS exposure may also engage endogenous anti-inflammatory pathways and blunt the anti-inflammatory effects of VNS. This work highlights the potential of VNS to prevent inflammation-induced hyperexcitability via complex, sex-dependent neuroimmune and other effects.

## Introduction

The physiological effects of electrical stimulation or interruption of the vagus nerve have been studied for over a century, and clinical vagotomy was once the preferred surgical treatment for peptic ulcer disease^1-4^. Early studies of vagus nerve stimulation (VNS) examined its effects on cardiac and gastric function, influences on brain activity, and later its ability to reduce seizures^1,2,4,5^. Today, VNS is a powerful electroceutical therapy with regulatory approval in several countries, including the United States. It is approved by the U.S. Food and Drug Administration (FDA) for the treatment of refractory epilepsy^6^, treatment-resistant depression^7^, cluster headaches^8,9^, migraines^10^, obesity^11^, and rheumatoid arthritis^12^, as well as improving outcomes during ischemic stroke rehabilitation^13^. Ongoing research and clinical trials are also exploring its effectiveness in other cardiovascular^4,14-16^, neurological^17-21^, gastrointestinal^3,22,23^, and pain-related^24-26^ contexts.

The broad therapeutic indications of VNS are rooted in the widespread connectivity and mixed composition of the vagus nerve. Approximately 80% of vagal fibers are afferent, transmitting sensory information related to touch, pain, taste, and visceral sensation to the brain, while the remaining efferent fibers convey parasympathetic signals to muscles and glands in the heart, lungs, and gastrointestinal tract^2,27-29^. Consequently, the mechanisms underlying the therapeutic effects of VNS are complex and multifactorial, influencing both central and peripheral processes.

The initial development and primary clinical use of VNS for drug-resistant epilepsy highlight its ability to modulate neuronal excitability^30-32^ and seizure susceptibility^33,34^. Studies suggest that VNS achieves these effects through multiple mechanisms, including activation of afferent vagal fibers projecting to the nucleus tractus solitarius, with subsequent modulation of downstream brain regions such as the locus coeruleus and dorsal raphe nucleus^35-37^. These pathways lead to changes in neurotransmitter release (e.g., norepinephrine, serotonin, GABA) as well as neural plasticity^38,39^.

In 2002, Kevin Tracey introduced the concept of the “inflammatory reflex”, a vagus nerve–mediated pathway through which the brain senses and regulates peripheral immune responses^40^. This pathway is thought to operate primarily through efferent vagal fibers that release acetylcholine, thereby suppressing pro-inflammatory cytokine release from immune cells^40-42^. Although this phenomenon has been studied extensively, the FDA only recently approved SetPoint Medical’s VNS device for the treatment of rheumatoid arthritis, making it the first approved inflammatory indication^12,43^. Increasing evidence also suggests that the anti-inflammatory effects of VNS may contribute to its therapeutic efficacy in epilepsy^44,45^. VNS has been shown to modulate neuroinflammation; however, it remains unclear whether these effects are indirect (via suppression of systemic inflammation), direct (through central neural pathways influencing glial cells and immune signaling in the brain), or a combination of both^18,44,46-52^.

Neuroinflammation contributes to numerous neurological disorders and can disrupt normal brain function, for example by increasing epileptogenicity^45,53^. One clinical condition characterized by both systemic and central inflammation with increased seizure risk is sepsis^54,55^. Sepsis results in a severe and often life-threatening systemic inflammatory response and can be accompanied by neuroinflammation, including microglial activation/transformation, oxidative stress, and increased production and infiltration of pro-inflammatory cytokines in the brain^56-61^. It is also associated with heightened neural excitability and reduced seizure thresholds, making patients more prone to seizures^54,55,61-63^. Sepsis-related neuroinflammation is increasingly recognized as a contributor to cognitive and neuropsychiatric complications, underscoring the importance of modulating neuroimmune pathways to prevent both acute and long-term neurological sequelae^54,59,60^.

A commonly used experimental model for studying systemic inflammation-induced neuroinflammation is endotoxemia induced by bacterial components such as lipopolysaccharide (LPS). Investigating the effects of VNS in an animal model of LPS-induced sepsis may shed some light on its mechanisms of action with respect to both systemic and central immune modulation. Such work is critical for advancing our understanding of how neuromodulation may interrupt the feedforward cycle linking systemic inflammation, neuroinflammation, and neural dysfunction.

We previously developed a wirelessly-driven, current-limited, implantable neuromodulation device small enough to be sutured directly around the rat vagus nerve^64^. Using this device, we demonstrated that after a chronic implantation, VNS suppressed pro-inflammatory TNF-α and increased anti-inflammatory IL-10 serum levels following a single LPS injection^64^. Here, we investigate the efficacy of VNS as an antiepileptic intervention in the context of LPS-induced systemic and central inflammation and examine whether its therapeutic effects are mediated through suppression of inflammation. We utilize a rat model of sustained endotoxemia induced by a five-day LPS regimen, which produces a prolonged but non-lethal inflammatory state resembling some aspects of sepsis. Using a refined implantable neuromodulation device with improved stimulation safety and enhanced wireless powering range, we administer daily VNS and assess its effects on epileptogenicity and immune regulation in both the periphery and brain. Our findings show that LPS significantly increases peripheral and central inflammation and lowers seizure threshold. VNS modulates aspects of the peripheral immune response and increases seizure threshold, with a complex and unclear effect on neuroinflammation.

## Results

### A Next-Generation Implantable VNS Device

**Figure 1:**
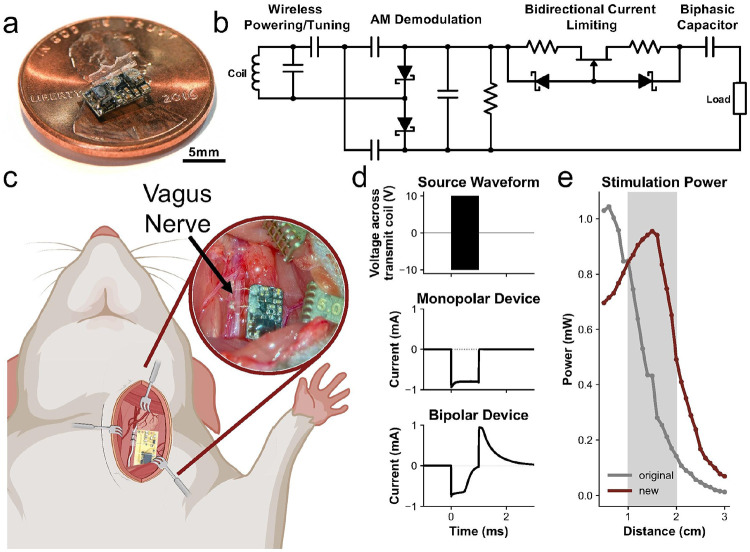
Next-generation, wirelessly-driven neurostimulator for VNS in rats. **a)** Image of our wirelessly-driven neurostimulation device on a U.S. penny. **b)** Passive stimulator circuit with impedance matching, full-wave voltage doubling rectifier for AM demodulation, bidirectional current limiting, and charge storage capacitor for charge-balancing. **c)** Rendering of the neurostimulator device being implanted in a rat (Created in BioRender. Lawlor, G. (2025) https://BioRender.com/nu13rxi) with inset showing a representative image of a surgically implanted VNS device. **d)** Examples of waveforms comparing the previous monophasic stimulation waveform to new, charge-balanced, biphasic stimulation waveform from the same transmitted amplitude-modulated radio-frequency signal **e**) Comparison between the first- and second-generation devices of power output as a function of distance between the transmit coil and device. The grey box highlights the usual operating distance between the device and transmit coil.

To facilitate the delivery of repeated VNS during a sustained inflammatory challenge, we enhanced both the stimulation safety and wireless powering range of our implantable neural stimulator^64^ by updating the passive circuitry (Fig. 1a,b). The device is small (5 × 7 × 1 mm) and can be sutured directly around the rat vagus nerve (Fig. 1a,c). It is powered wirelessly via electromagnetic coupling between an external transmit coil and receive coil located on the device. A pulse-modulated, radio-frequency signal is used to control stimulus delivery, defining the frequency, pulse width, and interphasic delay parameters. The amplitude is capped by the bidirectional current-limiting components as in Williams et al.^64^. Instead of a monophasic waveform, we introduced a charge-balanced, biphasic stimulus waveform by adding a series capacitor in-line with the load output and tuning the other passive values to create a fixed-return current waveform (Fig. 1d). This configuration prevents slow accumulation of charge at the electrode-tissue interface and irreversible electrochemical reactions that can result in electrode corrosion, degradation, and tissue damage^65^. Furthermore, to increase power efficiency, we implemented a full-wave voltage doubling rectifier instead of the simple half-wave rectifier. With these changes, we doubled the distance from which the device can receive the stimulation waveform and still reach 90% of peak wireless power transfer efficiency, allowing us to achieve the same current stimulus as the first-generation device from 0.7 cm farther away (Fig. 1e). The peak power transmission occurs at 1.5 cm, which is in the middle of the range of distances that we hold the transmit coil to apply VNS (Fig. 1e, gray band).

### LPS and VNS on Epileptogenicity

**Figure 2:**
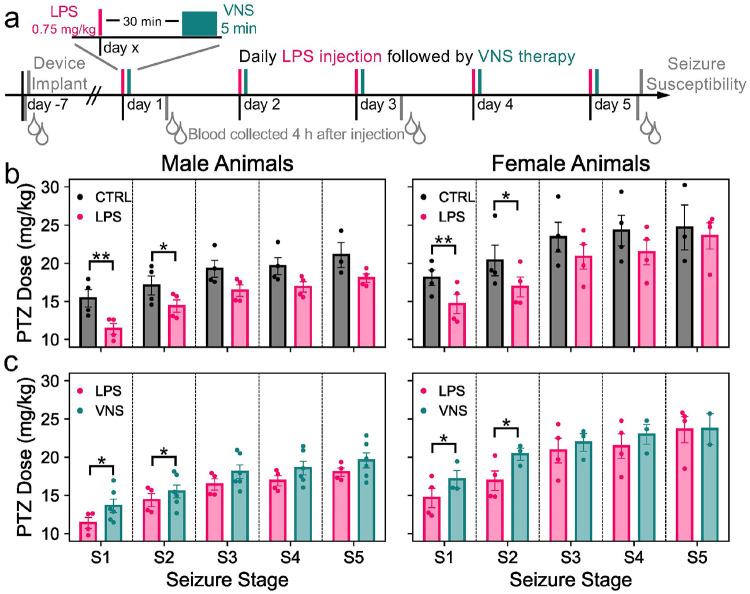
VNS counters heightened seizure susceptibility induced by LPS. **a)** Experimental timeline including device implantation, LPS injections and VNS therapy, blood collections, and the seizure susceptibility assessment. **b)** Pentylenetetrazol (PTZ) dose (mg/kg) that is required to achieve a designated seizure stage for control (black, n = 4 males & n = 4 females) versus LPS (pink, n = 4 males & n = 4 females) animals, grouped by sex. **c)** PTZ dose required to achieve each seizure stage for LPS (pink, same data in **(b)**) versus VNS (teal, n = 6 males & n = 3 females) animals, separated by sex. All data are presented as mean +/− standard error. Individual datapoints are overlaid. *p<0.05, **p<0.01.

Using our improved stimulation device, we first evaluated whether VNS could alter LPS-induced changes in epileptogenicity. Our experimental timeline is illustrated in Fig. 2a. Briefly, rats were implanted with a VNS device and, after a period of recovery, injected with LPS (0.75 mg/kg, i.p.) or saline daily for five days to induce sustained systemic inflammation^66^. Thirty minutes after each LPS injection, rats received VNS or sham stimulation (referred to as ‘shamVNS’) for 5 minutes. Blood for determining levels of inflammation markers was collected every other day from the lateral tail vein 4 hours after injection, and seizure susceptibility was assessed immediately after the final blood draw by intravenous infusion of pentylenetetrazol (PTZ). Animals receiving saline+shamVNS were designated as the control group, LPS+shamVNS the LPS group, and LPS + VNS the VNS group. Seizure progression stages, ranging from 1 to 6, were used to quantify the rate of progression of seizures and were based on our animals’ behavior and methods from studies publishing intraperitoneal and intravenous PTZ^67-69^. With this scoring, animals advanced through partial myoclonic jerks of the head and face to the body (stage 1), full myoclonic jerks rearing with both forelimbs (stage 2), full body convulsions (stage 3), head bowing (stage 4), tonic-clonic seizures (stage 5), and tonic limb extension (stage 6).

Animals in the LPS group showed a significant main effect of LPS treatment, with decreased seizure thresholds at stage 1 (F(1,12) = 12.5, p = 0.004) and stage 2 (F(1,12) = 4.83, *p* = 0.048) compared to control animals, indicating heightened seizure susceptibility with LPS treatment^67-69^ (Fig. 2b). PTZ dosage was decreased by 3.5 mg/kg in females for both stages and 4.0 mg/kg and 2.7 mg/kg for males at stages 1 and 2, respectively. Vagus nerve stimulation counteracted this, resulting in a significant main effect of VNS treatment and increased seizure thresholds at stages 1 and 2 in VNS animals compared to LPS animals (F(1,13) = 4.99, *p* = 0.044 and F(1,13) = 5.11, *p* = 0.042, respectively) (Fig. 2c). For stage 1, VNS increased PTZ dosage threshold by 2.4 mg/kg in females and 2.2 mg/kg in males. At stage 2, dosage increased by 3.5 mg/kg in females, matching the magnitude of the LPS-induced decrease, and 1.1 mg/kg in males. Furthermore, for all stages except stage 2 - LPS vs. control, the main effect of sex was significant (p < 0.05 – see Supplementary Data 1), with females displaying higher seizure thresholds than males. There was no significant interaction between treatment and sex (Supplementary Data 1).

### LPS and VNS on Peripheral Inflammation

**Figure 3:**
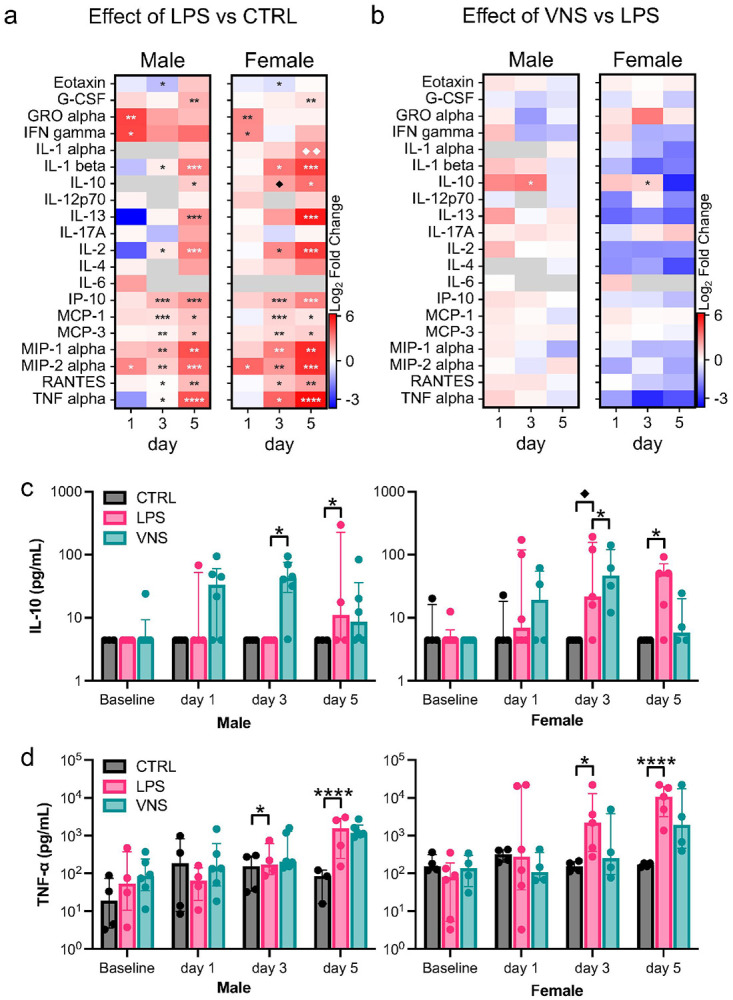
Cytokine immunoassays on serum samples. **a)** Heatmap of serum cytokine & chemokine differences between LPS (n = 4 males, n = 5 females) and control (n = 4 males, n = 4 females) animals 4 hours after LPS or saline injection **b)** Heatmap of differences between VNS (n = 6 males, n = 4 females) and LPS animals 4 hours after LPS injection. Cytokines are shown as the log_2_ fold change between group medians. Cytokines with readings below background are shown as grey. **c-d)** Anti-inflammatory cytokine IL-10 **(c)** and pro-inflammatory cytokine TNF-a **(d)** concentrations for each experimental group (control in black, LPS in pink, VNS in teal). Data are displayed as median +/− interquartile range. *p<0.05, **p<0.01, ***p<0.001, ****p<0.0001 of main effect of treatment by two-way ANOVA, ¨p<0.05, ¨¨p<0.01 by sex-stratified Student t-test following a significant interaction in the two-way ANOVA.

We hypothesized that the protective effects of VNS against LPS-induced epileptogenicity may be at least partially mediated by the suppression of peripheral and central immune responses. We previously showed that VNS reduced the peak TNF-α concentration and increased the peak IL-10 concentration after a single LPS challenge^64^. To assess the anti-inflammatory properties of VNS in our model, we analyzed cytokine concentrations in serum collected 4 hours after every other LPS injection. Lipopolysaccharide treatment produced a robust and progressive increase in multiple pro- and anti-inflammatory cytokines in the blood compared to control animals, confirming the induction of systemic inflammation in both male and female rats (Fig. 3a). On day 1, we observed a significant neutrophil-oriented response (GRO-α and MIP-2α) as well as an increase in IFN-γ. On day 3, there was a broader inflammatory and chemokine surge (IL-1β, IL-2, IP-10, MCP-1/3, MIP-1/2α, RANTES, and TNF-α) that persists on day 5 with the addition of an increase in the anti-inflammatory cytokines IL-10 and IL-13. Female rats tended to have a more pronounced response, with the main effect of sex being significant for 11 cytokines on days 3 and 5 (Supplementary Data 1). Full cytokine profiles across each timepoint can be found in Supplementary Fig. 1.

A significant main effect of VNS was observed for anti-inflammatory cytokine IL-10 (F(1,15) = 5.12, p = 0.039), indicating elevated levels in both sexes on day 3 (Fig. 3b,c). There was no significant suppression of pro-inflammatory cytokines at any time point, but in females, there was a general trend of reduction in levels of cytokines and chemokines, including, for example, TNF-α (Fig. 3d).

**Figure 4:**
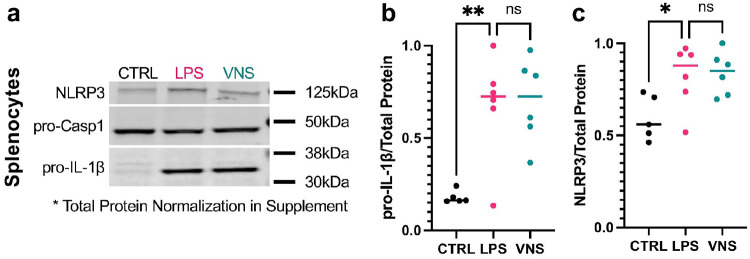
Western blotting results from splenocytes in all male cohort. **a)** Representative Western blots of NLRP3, pro-Caspase1, and pro-IL-1β. **b-c)** LPS results in an increased expression of (**b)** pro-IL-1β and **(c)** NLRP3 in splenocytes. Pro-Caspase1 was not significantly changed. VNS did not show significant changes from the LPS group. All data are presented as median with individual datapoints. **p<0.01, ***p<0.001, ns=nonsignificant.

We also performed a Western blot analysis on splenocytes for key inflammatory proteins involved in LPS-induced inflammation shown to be modulated by VNS^70-72^ from an independent male cohort. We found that NLRP3 and pro-IL-1β were significantly upregulated in LPS-treated animals compared to controls but did not detect significant differences between LPS and VNS groups (Fig. 4).

**Figure 5:**
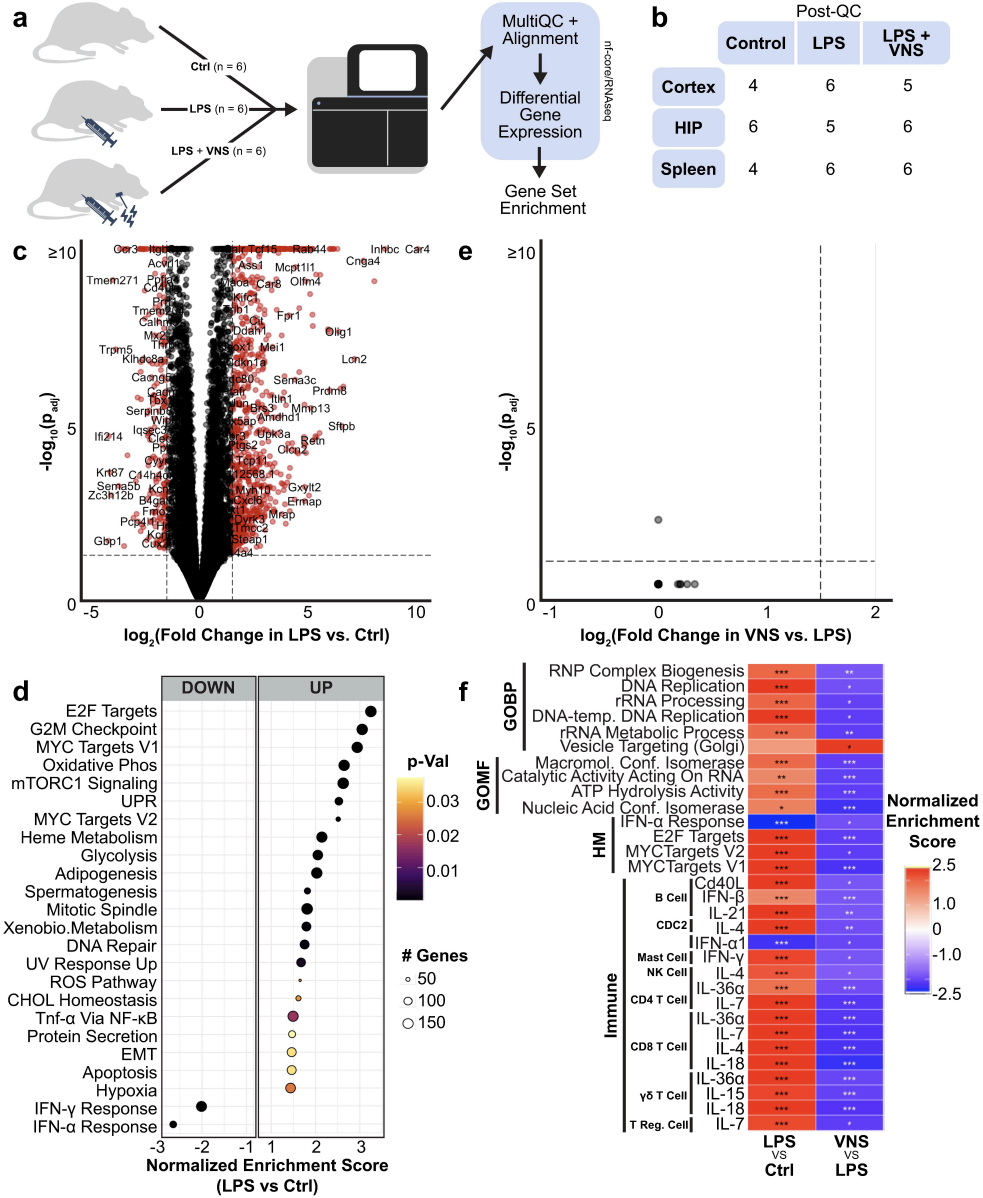
RNA-sequencing analysis of splenocytes. **a)** An independed cohort of 18 male animals (n = 6 per group) were treated with LPS+shamVNS (LPS), LPS+VNS (VNS), or naïve (Ctrl). Cortex, hippocampus, and splenocytes from the animals were sequenced. **b)** Post quality control (QC) animals for RNA-Seq analysis excluded 2 Ctrl and 1 VNS cortex sample, 1 LPS hippocampus sample, and 2 spleen Ctrl samples due to quality issues such as high levels of read duplication. **c)** Volcano plot of differential gene expression in splenocytes after LPS treatment as compared to naïve. Significantly changed genes are marked with a red circle (p_adj_ < 0.05, log_2_FoldChange > 1.5). **d)** Hallmark gene sets that are significantly (p_adj_ < 0.05) enriched in the splenocytes after LPS treatment as compared to naïve. **e)** Volcano plot of differential gene expression in spleen after LPS+VNS treatment as compared to LPS only. **f)** Gene gene ontology (GO) biological process (BP) and molecular function (MF), Hallmark (HM), and Immune gene sets that are significantly (p_adj_ < 0.05) enriched in the splenocytes after LPS+VNS as compared to LPS only and the corresponding pathways from the LPS vs. naïve gene set enrichment analysis. *p<0.05, **p<0.01, ***p<0.001.

The effects of LPS and VNS were also explored with RNA sequencing (RNA-Seq) of splenocytes from the same experiment (Fig. 5a,b). The effect of LPS revealed many differentially expressed genes (DEGs) with 782 upregulated and 128 downregulated in the LPS group compared to controls (Fig. 5c). Differential expression and gene set enrichment analyses comparing LPS-treated animals versus controls revealed broad transcriptional remodeling consistent with activation of innate inflammatory and antimicrobial defense responses, coupled with the induction of regulatory and reparative programs. Enrichment of proliferative and metabolic pathways (including G2M checkpoint, E2F/MYC targets, oxidative phosphorylation, and mTORC1 signaling) indicated increased cellular turnover and biosynthetic activity. Conversely, type I interferon, T cell activation, and adaptive immune response pathways were downregulated, consistent with immune tolerance and resolution. Analysis of cell type–specific immunologic gene set signatures^73^ further indicated coordinated activation of cytokine response pathways driven by both pro- and anti-inflammatory mediators, alongside suppression of type I interferon–associated programs.

We then compared the transcriptomic profiles of LPS+shamVNS and LPS + VNS animals. Although no individual transcripts reached significance, GSEA revealed reversal of several pathways upregulated by LPS. VNS downregulated proliferative and metabolic programs (including E2F/MYC targets, DNA replication, and ribosome biogenesis) and further suppressed type I interferon signaling, consistent with attenuation of sustained inflammatory activation. Immunologic signature analysis also indicated reduced responsiveness of B and T cells to cytokine stimulation. Together, these results suggest that VNS may result in shifts towards immune quiescence and re-establishment of normal cellular activity in the spleen. For a more detailed analysis of transcriptomic changes, see Supplementary Analysis 1.

### LPS and VNS on Central Inflammation

Next, we explored the central effects of repeated LPS treatment and to what extent these are corrected by VNS^46,47,63,74^. To this end, we assessed different brain regions for morphological evidence of microglial activation, a hallmark of neuroinflammation^75,76^, as well as for changes in select inflammatory proteins.

**Figure 6:**
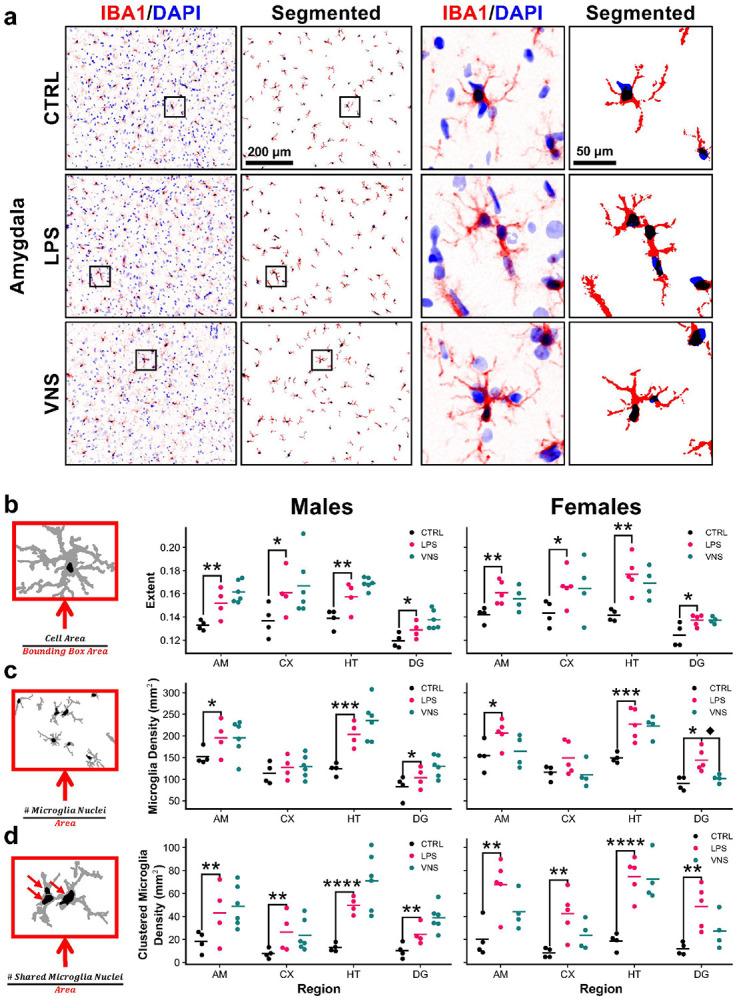
Immunohistochemistry of microglia cytology shows strong LPS-treatment. **a)** Representative confocal images of IBA1(+) microglia (red) and DAPI(+) nuclei (blue) in the amygdala (AM) in female rats. Confocal images with enhanced contrast to highlight processes followed by images of segmented microglia used for analysis. Further plots indicate a magnified area. **b-d)** Plots of microglial cytology measurements, namely **(b)** *bounding box* extent, **(c)** microglia density, and **(d)** clustered microglia density for control (black), LPS (pink), and VNS (teal) animals, separated by sex in the amygdala (AM), cortex (CX), hypothalamus (HT), and dentate gyrus (DG). All data are presented as mean with individual datapoints. *p<0.05, **p<0.01, ***p<0.001, ****p<0.0001 of main effect of treatment by two-way ANOVA, ¨p<0.05 by sex-stratified Student t-test following a significant interaction in the two-way ANOVA.

Immunohistochemistry for IBA1, a calcium-binding protein specific to microglia and macrophages, shows that LPS treatment alters microglial cytology relative to controls in amygdala (Fig. 6a). Representative images for the dentate gyrus, hypothalamus, and cortex are shown in Supplementary Figs. 2–4. Microglial profiles were quantified based on the bounding box extent (a ramification metric), microglia density, and clustered microglia density. Lipopolysaccharide significantly increased the extent and clustered density in all four regions, and microglial density was increased in the amygdala, hypothalamus, and dentate gyrus (Supplementary Data 1) (Fig. 6b-d). Vagus nerve stimulation did not reverse these metrics in males, but in females it led to a reduction in microglial density in the dentate gyrus (t(7) = 3.11, p = 0.017) following a significant interaction effect (F(1,15) = 10.78, p = 0.040). There was also a trend toward reduced microglia density and clustered microglia density in the amygdala, cortex, and dentate gyrus with VNS treatment.

**Figure 7:**
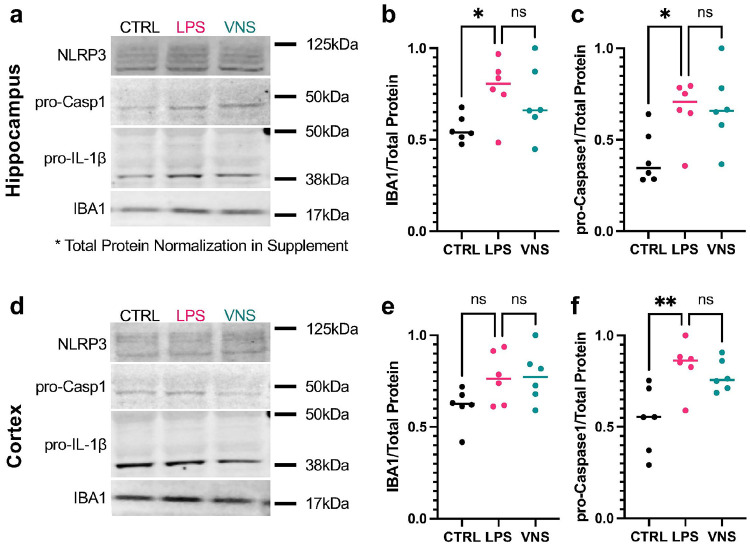
Western blotting results from the brain in all male cohort. **a)** Representative Western blots from the hippocampus of NLRP3, pro-Caspase1, and pro-IL-1β, and IBA1. **b-c)** LPS results in an increased expression of (**b)** IBA1 and **(c)** pro-Caspase1 in the hippocampus. **d)** Representative Western blots from the frontal cortex of NLRP3, pro-Caspase1, and pro-IL-1β, and IBA1. **e-f)** LPS did not result in an increased expression of (**e)** IBA1 but did significantly increase **(f)** pro-Caspase1 in the frontal cortex. VNS did not show significant changes from the LPS group. NLRP3 and pro-IL-1β were not significantly changed in either region. All data are presented as median with individual datapoints. *p<0.05, **p<0.01, ns=nonsignificant.

Western blot analysis showed an increase in IBA1 protein in the hippocampus, consistent with the increase observed in microglia density. Pro-caspase-1 was increased in both the hippocampus and frontal cortex. NLRP3 and IL-1β did not show significant differences. There were no significant differences between LPS and VNS groups (Fig. 7).

**Figure 8:**
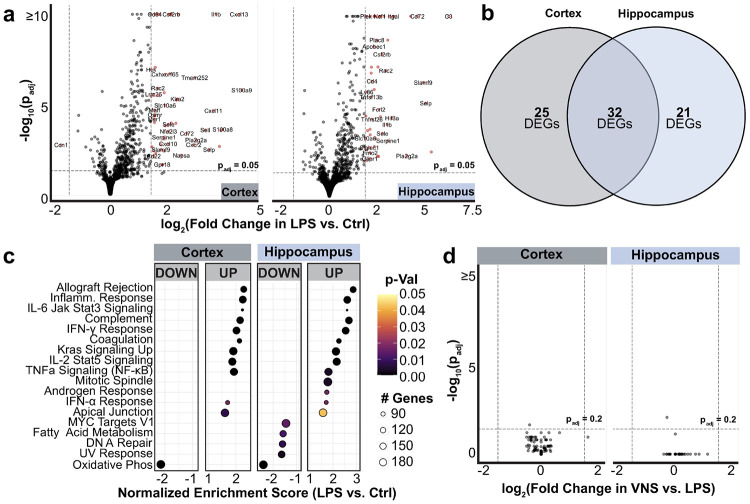
RNA-Sequencing analysis of brain regions. **a)** Volcano plots of differential gene expression in cortex (left) and hippocampus (right) after LPS treatment as compared to control. Significantly changed genes are marked with a red circle (p_adj_ < 0.05, log_2_FoldChange > 1.5). **b)** 32 upregulated genes after LPS treatment are shared between cortex and hippocampus. **c)** Hallmark gene sets that are significantly (p_adj_ < 0.05) enriched in the cortex (left) and hippocampus (right) of the LPS group as compared to Ctrl. **d)** Volcano plots of differential gene expression in cortex (left) and hippocampus (right) of the VNS treatment group as compared to LPS only.

RNA sequencing showed that LPS significantly upregulated 57 genes in the cortex and 53 genes in the hippocampus (Fig. 8a). Thirty-two of these genes overlap between the two regions (Fig. 8b) and are related to innate immune activation and inflammation, immune cell migration and adhesion, and negative regulation of immune signaling. Similarly, GSEA revealed upregulation of inflammatory, complement, and microglial activation pathways. Concurrently, metabolic and biosynthetic programs, such as oxidative phosphorylation and ribosome biogenesis, were suppressed. Notably, pathways associated with anti-inflammatory and resolutory mechanisms, IL-10/IL-4/IL-13 signaling, apoptotic cell clearance, and negative regulation of adaptive immunity and NF-κB activity, were also enriched. These findings indicate that sustained LPS exposure triggers a robust pro-inflammatory response in the brain while simultaneously activating programs that may limit tissue injury and promote resolution.

Treatment with VNS did not significantly alter the transcriptional response to sustained inflammation, as determined by the lack of significant DEGs or enriched pathways from the Hallmark, GO, or Immunologic Signature gene sets. However, we also investigated a curated database for brain-related functional gene sets (Brain.GMT)^77^ which revealed selective effects in the cortex but not hippocampus (Supplementary Fig. 5). These included a significant downregulation of multiple oligodendrocyte-related pathways and gene sets known to be upregulated in stress-susceptible animals vs. stress-resilient animals, perhaps reflecting a shift toward a “stress-resilient” transcriptional profile. For a more detailed analysis, see Supplementary Analysis 2.

## Discussion

This study addresses the role of VNS in modulating epileptogenicity in the context of systemic inflammation, with a particular focus on its purported anti-inflammatory effects^40^. This area has been relatively underexplored, with prior literature primarily concerned with antiepileptic effects in classical rodent kindling models, focusing on mechanisms such as neuroplasticity^38,39,78^, neurotransmitter changes^38,79^, and desynchronization of neural circuits^30^. Our findings demonstrate that VNS mitigates the heightened seizure susceptibility induced by sustained inflammation. Using an improved implantable VNS device capable of safe, charge-balanced stimulation, we showed that daily stimulation following LPS administration increases seizure threshold, elevates peripheral levels of the anti-inflammatory cytokine IL-10, and suppresses proliferative, biosynthetic, and inflammatory activity of peripheral immune cells at the transcriptomic level. However, VNS did not appear to influence LPS-associated neuroinflammatory responses at the cytological, biochemical or transcriptomic level. These results suggest that while VNS corrects LPS-induced epileptogenicity, its effects may operate via central mechanisms distinct from a classical suppression of neuroinflammation.

### LPS-Induced Inflammation and Epileptogenicity

It is well established that acute LPS exposure induces an intense systemic inflammatory response, but the consequences of daily LPS injections are not as defined. Some studies show that repeated LPS administration produces a peripheral inflammatory response that can evolve toward a state of endotoxin tolerance, marked by reduced pro-inflammatory cytokine release and compensatory upregulation of anti-inflammatory mediators such as IL-10^66,80,81^. Other reports indicate that tolerance is not always achieved, with persistent elevation of pro-inflammatory cytokines and sometimes no increases in anti-inflammatory cytokines^63,82^.

Our findings reflect an intermediate phenotype. On the first day of LPS treatment, we observed a strong neutrophil-oriented response consistent with acute exposure, including elevations in GRO-α, MIP-2α, and IFN-γ. By days 3 and 5, elevated cytokine and chemokine profiles included IL-1β, IL-2, TNF-α, MCP-1/3, MIP-1/2α, IP-10, and RANTES, accompanied by progressive increases in IL-10 and IL-13. This evolving pattern suggests that pro- and anti-inflammatory responses can co-occur during repeated endotoxin challenges, with partial engagement of regulatory cytokines that may temper, but not fully resolve, the inflammatory cascade. Differences from prior reports may relate to lower LPS dose (0.75 mg/kg vs. 0.5 mg/kg) and the use of weaker serotypes (O111:B4 vs. O55:B5 and O26:B6), which could elicit different inflammatory responses^82-84^. We also calculated the dose based on animal weight from day 1 and did not adjust for weight changes. Since all LPS-treated animals lost weight (Supplementary Fig. 6), this may have effectively increased the relative dose over time, potentially explaining the partial engagement of tolerance mechanisms in our model^85^.

Western blot analysis of splenocytes confirmed activation of innate inflammatory pathways, with upregulation of NLRP3 inflammasome and its downstream effector pro-IL-1β. Transcriptomic profiling revealed strong upregulation of innate and antimicrobial genes and tissue-repair mediators, alongside anti-inflammatory regulators, whereas type I interferon and adaptive immune genes were downregulated. GSEA confirmed enrichment of proliferative and metabolic pathways, with suppression of interferon and adaptive responses. Together, these data depict a metabolically active, yet partially tolerant, immune state.

In the CNS, sustained systemic inflammation induced microglial transformation and clustering in multiple brain regions, indicating neuroinflammation. This is consistent with observations by Huffman et al. and Kim et al., who observed increases in unramified, hypertrophic, microglia^46,66^. Elevated IBA1 in the hippocampus and pro-caspase-1 in the hippocampus and cortex align with the measured increase in microglia density^47^. Transcriptomic analyses revealed induction of innate immune and cytokine signaling genes and enrichment of inflammatory pathways, such as Allograft Rejection, IL6/JAK/STAT3 Signaling, Complement, and Interferon-γ Response. Yet, resolution-associated programs, including IL-10, IL-4, and IL-13 production, complement regulation, and apoptotic cell clearance, were also upregulated, suggesting that neuroinflammatory activation is accompanied by local regulatory or reparative processes aimed at limiting damage. These findings are consistent with a prior report of early, mixed inflammatory and homeostatic responses in the CNS following sustained immune stimulation^66^. For a detailed discussion of transcriptomics, see Supplementary Analysis 1.

Our results confirm prior evidence that systemic inflammation induced by LPS decreases seizure thresholds and promotes neuronal hyperexcitability^62,86-88^. LPS treatment significantly increased susceptibility in stage 1–2 seizures (characterized by myoclonic jerking), with non-significant increases in stage 3–6 seizures. This contrasts with other studies using different LPS dosing regimens, typically a single dose within 24 hours or other kindling models^62,63,89^, that reported significant decreases in seizure threshold for stage 5–6 seizures. The reasons for these stage-specific differences remain unclear but may relate to variations in seizure induction protocols, magnitude of systemic inflammation, or region-specific neuroimmune responses.

### VNS and Antiepileptic Effects in the Context of LPS-Induced Inflammation

VNS effectively counteracted the epileptogenicity caused by sustained inflammation, primarily by increasing seizure threshold at stages 1 and 2. This aligns with previous clinical and preclinical findings showing that VNS reduces seizure frequency and severity, but it is the first demonstration of this effect in a systemic inflammatory model. The absence of clear anti-neuroinflammatory evidence suggests that anti-seizure effects may be mediated by non-inflammatory mechanisms, consistent with acute anti-seizure effects observed in classical epileptic models^34^.

Our VNS protocol did not suppress the repeated LPS-induced elevations in pro-inflammatory cytokines, contrasting some prior studies demonstrating that VNS reduces systemic TNF-α 1–3 hours after a single-dose LPS challenge^41,46,64,90^. It is possible, however, that by our 4-hour sampling time, TNF-α signaling had already peaked and declined, masking early modulatory effects on day 1. On the other hand, VNS increased IL-10, most evidently on day 3, suggesting that its inflammatory effects may be short-lived or overwhelmed by daily LPS doses. Although not evident at day 5, this transient IL-10 elevation could still contribute to the increased in seizure threshold, consistent with reports linking IL-10 to neuroprotection and anticonvulsant effects^91,92^.

At the transcriptional level, VNS did not induce significant differential gene expression but altered pathway-level signatures. GSEA revealed reversal of LPS-induced proliferative and metabolic programs and suppression of adaptive cytokine responses, indicating a shift toward a less inflammatory, more metabolically quiescent state. These findings suggest that the longer-term therapeutic effects of VNS may reflect restoration of immune homeostasis rather than direct inhibition of cytokine release (see Supplementary Analysis 2 for more details).

Cytological and biochemical markers of neuroinflammation were not reversed by VNS, although trends toward decreased microglia density and clustering were observed in females, with a significant reduction in the dentate gyrus. This contrasts with reports showing VNS reduces microglial activation after a single LPS dose^46,47^, possibly due to our repeated LPS dosing or differences in VNS parameters (our study: 5 Hz, 500 μs pulse width, 1 mA biphasic pulses; Meneses et al.: 5 Hz, 2000 μs, 0.75 mA; and Huffman et al.: 10 Hz, 300 μs, adjustable amplitude with needle electrode)^46,47^. Vagus nerve fiber recruitment is highly dependent on stimulation parameters^93,94^, and even Huffman et al. found that while 10 Hz stimulation caused a successful restoration of ramified microglia, 20 Hz stimulation did not^46^. Overall, there were trends of the anti-inflammatory effect of VNS, but it was unable to restore inflammation to control levels.

There are few studies on VNS with transcriptomics or proteomics in any disease^18,95-98^. In epilepsy patients, VNS downregulated stress, inflammatory, and immune-related genes in blood^95^, paralleling our findings of peripheral immunomodulation. In a model of multiple sclerosis^18^ and learning/memory^98^, minimal CNS differential gene/ protein expression was observed, but pathway-level changes occurred in synapse-related pathways, specifically glutamate-related pathways, positive regulation of myelination, downregulation of mature oligodendrocyte protein, as well as reduced stress signaling. Consistent with these findings, our data from the cortex show limited differential expression but pathway-level shifts, including downregulation of multiple oligodendrocyte gene sets as well as a shift toward a stress-resilient transcriptional profile, which may indicate similar effects of VNS across distinct models^18,98^.

### Issues Related to Sex Dimorphism

Sex differences were observed, with females displaying higher seizure thresholds and elevated levels of inflammatory markers, consistent with the known effects of sex hormones on seizure susceptibility^99-101^ and inflammation^102,103^. VNS treatment exhibited a reduction trend in females only, including pro-inflammatory cytokines (IL-1α, IL-1β, IL-2, and TNF-α) and microglial activation markers in the amygdala, cortex, and dentate gyrus, with dentate gyrus microglia density reaching significance.

This result may owe to the fact that cytological markers of microglial activation are stronger in females than males treated with LPS, making the anti-inflammatory effects of VNS more apparent. We also cannot rule out the possibility that the 100-g smaller, age-matched females may have received different VNS stimulation amplitudes due to shorter distance or less tissue between the powering coil and stimulation device, since males were larger and had more subcutaneous fat; however, this distance was within the range of peak power transfer efficiency for our VNS devices (Fig. 1e) and current-limited to 1 mA, making this explanation unlikely. Sex dimorphism in VNS effects is important and warrants further investigation, especially given the growing interest in clinical VNS therapeutics.

### Implications for Anti-Inflammatory VNS-Treatment

These results suggest that after sustained inflammatory exposure, the immune system, while still exhibiting significant inflammatory activity, begins engaging endogenous resolution mechanisms that limit inflammation and promote repair. This transition is often marked by reduced IFN-γ and elevated IL-10^104^, a pattern we observed after five consecutive daily LPS doses (Fig. 3a). In otherwise healthy animals, two resolution mechanisms may occur via the vagus nerve: (1) the fast-acting cholinergic anti-inflammatory reflex and (2) a slower-acting afferent-hypothalamic pathway inducing anti-inflammatory adrenocorticotropic hormone and glucocorticoid release^40^. Supporting this, repeated intratracheal LPS with left-sided vagotomy increased lung inflammation severity, suggesting intact vagal pathways contribute meaningfully to resolution^105^.

Because endogenous resolution mechanisms appear engaged from day 3, the incremental effect of daily VNS may be overshadowed. To our knowledge, no other studies have examined anti-inflammatory effects of VNS in sustained LPS paradigms, raising the possibility that VNS may not modulate this inflammatory reaction to the same extent as it does acute inflammatory responses. This hypothesis is supported by a 2024 meta-analysis of human VNS clinical trials, reporting no consistent anti-inflammatory effect overall, but acute inflammatory states (e.g., sepsis, surgery) showed reduced C-reactive protein^106^. Therefore, if endogenous vagal activity is already upregulated during a sustained inflammatory challenge, exogenous VNS may provide limited incremental benefit. Nevertheless, VNS may still be of therapeutic value in conditions with reduced vagal tone such as in heart failure, irritable bowel syndrome, and depression^107^.

### Limitations and Future Directions

First, the relatively short duration of VNS treatment may have limited detectable anti-inflammatory effects, especially in the brain. Second, VNS was administered under isoflurane, which may confound anti-inflammatory outcomes^108,109^. Future work will focus on wearable devices enabling VNS in awake, freely moving animals. Third, optimizing VNS parameters may improve inflammatory modulation. Patient-specific tuning using real-time biomarkers, such as endogenous vagal tone, may help identify optimal therapeutic windows^45,110^. Understanding the baseline vagal tone in control and LPS animals might help in understanding which inflammatory states are best treated by VNS. Fourth, selective efferent or afferent VNS could clarify mechanism by which the inflammatory reflex affects epileptogenicity. Finally, extending studies to chronic inflammation models, including traumatic brain injury, will provide insights into VNS effects beyond sustained endotoxemia.

## Methods

### VNS Device Manufacturing and Testing

Our new device, shown in Fig. 1a, is populated and packaged as described in Williams et al., which also includes information on cuff fabrication^64^. For these experiments, a signal generator (N5172B, Agilent Technologies, Santa Clara, CA, USA) was used to generate the pulse-modulated, radio-frequency signal to drive the stimulator. This signal was amplified by a power amplifier (ZHL-1-2W-S+, Mini-Circuits) to achieve an output power of 1 W. A tuned transmit coil (2 turns of 22 AWG enameled magnet wire with a diameter of 18 mm) was used to inductively couple the signal to the receive coil located on the device.

Unlike in Williams et al., a 1.2 mA I_DSS_ JFET was utilized with 390 Ω resistors to set the current limit at 1.0 mA, determined by inputting a 100 Hz, 10 V_PP_ sinusoid and measuring the voltage across a 1 kΩ load resistor^64^. Powering distance was assessed by measuring the current across a 1 kΩ load resistor while powering the devices from various distances. To find the difference in overall powering range between the two devices, the new device’s power versus distance curve was shifted on the x-axis by a distance x_0_, then the x_0_ that resulted in the least-squared error between the original and new device with a step size of 0.1 cm was found to be 0.7 cm (mean squared error of 0.056 cm^2^, compared to values greater than 2 cm^2^ at poorly shifted x_0_). The range of realistic rat VNS powering distances of 1–2 cm was found by measuring the depth of the vagus nerve beneath the skin, normally less than 1.4 cm, and adding some distance to account for the coil being held just off the skin.

### Animal Experiments

Twelve-week-old Long Evans rats (*n* = 45; males (M): *n* = 32, 337 ± 26 g; females (F): *n* = 13, 235 ± 14 g) (Inotiv, Lafayette, IN, USA) were used in this study. Both sexes were included in the first experiment to account for sex as a biological variable. Animals were subjects in one of two experiments described below. In Experiment 1, we investigated the effects of LPS and VNS on peripheral and central inflammation and seizure susceptibility (*n* = 30; 14M, 16F); animals were divided into the saline+shamVNS group, designated as the control group (*n* = 8; 4M, 4F), LPS+ShamVNS group, designated as the LPS group (*n* = 11; 4M, 5F), and LPS-VNS group, designated as the VNS group (n = 11; 6M, 4F). In Experiment 2, we investigated the effect of VNS on LPS-treated animals using Western blotting and transcriptomics (*n* = 18; 18M). Animals were divided into naïve (*n* = 6), LPS+shamVNS, designated the LPS group (*n* = 6), and LPS-VNS, designated the VNS group (*n* = 6). These numbers are prior to any exclusions due to mortality or experimental error, which will be detailed in related sections.

Animals were housed with a 12-hour light/dark cycle with *ad libitum* access to food and water. All procedures were performed in accordance with the Johns Hopkins University Institutional Animal Care and Use Committee.

### VNS Device Implantation

Animals were induced with 5% inhaled isoflurane mixed with oxygen and maintained with 1.5–2.5% isoflurane mixed with oxygen at 2 L/min. Preoperative doses of Ethiqa XR (NDC 86084-100-30, Fidelis Animal Health, New Jersey, USA) at 0.65 mg/kg were injected subcutaneously to ensure appropriate analgesia. Depth of anesthesia was monitored with the toe-pinch reflex, respiration rate, heart rate, and blood oxygenation. A constant body temperature of 36.5°C was maintained using a closed-loop heater with rectal thermometer (PY2 50-7212, Harvard Apparatus, Holliston, MA, USA). Vagus nerve devices were implanted as in Williams et al.^64^. Briefly, the neck was shaved, sterilized with alternating swabs of chlorhexidine and 70% isopropyl prep pads, and covered with a sterile drape. A 15 mm incision was made on the neck, parallel to the trachea, roughly 2mm lateral to the midline on the animal’s left side. The connective tissue and glands were bluntly dissected and the sternohyoid, omohyoid, and sternomastoid muscles were retracted to expose the carotid sheath. Approximately 10 mm of the vagus nerve below the carotid bifurcation was separated from the carotid artery and placed inside the cuff of the device. To secure the nerve, two 6 – 0 silk sutures were tied around the parylene cuff to close it. The device printed circuit board was positioned below the sternomastoid muscle, and the muscles were carefully released over it. The incision was closed with 4 – 0 polyglycolic acid sutures and coated with triple-antibiotic ointment.

### LPS Injections

A week after implantation, subjects in the LPS and VNS groups received intraperitoneal injections of LPS from *E. coli*, serotype O111:B4 (L2630, Sigma-Aldrich, St. Louis, MO, USA), at 0.75 mg/kg in saline every 24 hours for five days. The dosage was determined based on the animal’s weight on day 1 and maintained constant throughout the experiment. Animals were briefly anesthetized with 5% isoflurane for 1.5 minutes to administer the injection. The LPS was supplied as a lyophilized powder and reconstituted in sterile saline to a final concentration of 2.5 mg/mL, then vortexed for 15 minutes, aliquoted, and stored at −80°C until use. Based on our experience, the lethal dose 50% (LD50) of a single, IP injection of LPS reconstituted by vortexing is 5 mg/kg, but after sonication, the LD50 is ~ 1 mg/kg. This experiment utilized 2 separate bottles of LPS, one for each experiment. Animals in the control group received saline alone. Naïve animals did not receive injections.

### VNS Therapy

Thirty minutes after LPS/saline injections, all animals were re-anesthetized with isoflurane for VNS vs. shamVNS therapy. Animals were induced at 5% inhaled isoflurane for 2 minutes before transferring to a nose cone at 2.0% isoflurane to receive VNS for 5 minutes. The transmit coil was aligned with the implanted coil to the best ability of the experimenter. The coil was positioned as close to the animal’s neck as possible without touching and held in place by a flexible-arm clamp. The signal generator (N5172B, Agilent Technologies) was configured to produce a 27.12 MHz sine wave pulse-modulated at 5 Hz with a 1 ms on-time. The resulting stimulation waveform was a charge-balanced, bipolar stimulation pulse with a pulse width of ~ 500 μs and an inter-pulse delay of ~ 500 μs. Successful device activation was confirmed by 5 Hz muscle contractions in the neck, an off-target effect.

### Serum Collection

Blood was collected from the lateral tail vein of restrained animals 4 hours after LPS/saline injection before device implantation and on the 1st, 3rd, and 5th days of injections/therapy. Approximately 200 μL of blood was collected and allowed to clot at room temperature (RT) for 20 minutes before centrifuging at 2000 × g for 10 minutes at RT. Then the supernatant (serum) was stored at −20°C until analysis.

### Seizure Susceptibility Assessment and Analysis

On the 5th day of injections/therapy, the animals were acclimated for 1 hour in a BASi Universal Cage located on a Raturn System spinning base (MD-404 & RT-203, BASi, West Lafayette, IN, USA) to allow for free movement during infusion. During this time, pentylenetetrazol (PTZ) (P6500, Sigma-Aldrich) was freshly dissolved into sterile saline at 10 mg/mL. A microsyringe pump (14-831-200, Fisher Scientific, Waltham, MA, USA) set to dispense at a rate of 1 mL/min was used to infuse the PTZ solution until the animal reached a generalized tonic-clonic seizure, or until 1 minute of infusion had elapsed. Video was recorded from before the start of infusion until after infusion stopped and seizure progression had halted.

Recorded videos were annotated using the ELAN software (version 6.8) by a blinded experimenter who graded the seizure progression based on stages: 1 - myoclonic jerking of the head and face to the body, 2 – myoclonic jerking involving rearing with both forelimbs, 3 – convulsion of entire body, 4 – transition to tonic-clonic seizure characterized by head bowing, 5 – whole body tonic-clonic seizure (usually on belly), and 6 – forelimb/hindlimb extension. These seizure stages were derived from observations of the animals’ seizure progression as well as publications of intraperitoneal and intravenous PTZ seizure stages^67-69^. Two animals were excluded: one bit a hole in the tubing during infusion causing immeasurable leakage and another reached the maximum infusion time without evidence of seizure, and it was unclear if the catheter was properly placed in the vein.

### Transcardial Perfusion and Immunohistochemistry

Immediately following the seizure susceptibility assessment, animals were deeply anesthetized with 100–200 mg/kg Euthasol and transcardially perfused with 37°C 1× PBS to remove the blood then freshly depolymerized 4% paraformaldehyde for 15 minutes at a rate of 27 mL/min. The brain was post-fixed in the same fixative at 4°C for 24 hours, cryoprotected (5% DMSO, 20% glycerol), and stored at 4°C. Frozen brains were sectioned coronally at 40 μm using a sliding microtome (HM 400, Microm, Heidelberg, Germany) and stored in antifreeze buffer (30% sucrose, PVP-40, ethylene glycol) at −20°C until staining. Three serial sections were selected from each animal between Bregma − 3.0 to −3.4 mm and processed for immunohistochemistry. Briefly, sections were blocked with 4% normal donkey serum and 0.4% Triton X-100 in TBS at RT for 1 hour and then incubated overnight at 4°C with rabbit anti-IBA1 (019-19741, 1:1000, Wako, Osaka, Japan). Following three rinses in TBS, the sections were incubated in donkey anti-rabbit AlexaFluor 594 Plus (A32754, 1:300, Invitrogen, Waltham, MA, USA) at RT for 4 hours. After two additional rinses in TBS with 0.1% Tween-20, sections were counterstained with DAPI (D21490, Invitrogen), mounted, and cover-slipped with Vectashield (H-100-10, Vector Laboratories, Newark, CA, USA). Sections were imaged using the MICA Leica microscope (11889180, Leica, Wetzlar, Germany) using a 20× objective lens. Confocal images of the hippocampal dentate gyrus and CA3, motor cortex, hypothalamic nucleus, and amygdala were acquired in a z-stack of 4 images, 2 μm apart.

### Microglia Analysis

Regions of interest were manually drawn using raw microglia and nuclei images, then microglia soma and whole microglia masks were created with smoothing filters in FIJI (version 2.14.0). In CellProfiler 4.2.7, nuclei and microglia were separately segmented with adaptive Otsu thresholding. To optimize the full capture of two-dimensional microglia images, thresholding parameters were manually adjusted to balance the inclusion of spotty microglia processes as continuous processes while minimizing the inflation of process thickness. Microglia were matched to nuclei and microglia with less than 100 μm^2^ in area or without nuclei were excluded, as these objects are likely incompletely captured microglia and fall out of the expected range of areas^111,112^. The bounding box extent is the cell area divided by the smallest possible bounding box area drawn around the microglia – a robust measure of microglia activation^113-115^ chosen to characterize microglia cytology. In the presence of inflammation, the length of processes decreases and their thickness increases, resulting in a larger microglia area and a smaller bounding box, therefore larger extent. Clusters of multi-nuclei microglia aggregates, typically seen in Alzheimer’s^116^, traumatically injured brains^117^, and after LPS injections^66^, were noted after LPS treatment and quantified by their density.

### Cytokine Immunoassay Analysis

Blood serum samples were analyzed using ProcartaPlex^™^ Rat Cytokine & Chemokine Panel, 22plex assays (EPX220-30122-901, Thermo Fisher Scientific, Waltham, MA, USA). The samples were analyzed in duplicate as per manufacturer’s instructions. Thermo Fishers Scientific’s ProcartaPlex Analysis App was used to fit the standard curve for each cytokine and remove data points with technical issues or low bead counts before exporting the data for statistical analysis. Supplementary Fig. 1 shows the cytokine concentrations for all tested cytokines with concentrations greater than the limit of detection.

### Transcardial Perfusion for Protein and RNA Methods

Three hours following the final LPS injection, animals were deeply anesthetized and maintained with 5% inhaled isoflurane mixed with oxygen at a rate of 2 L/min. The spleen was quickly removed for splenocyte isolation, and the animal was transcardially perfused as previously described, but only with 4°C 1× PBS for exactly 30 seconds to remove blood without flushing out all excreted proteins. The brain was removed and processed over ice for protein and RNA extraction. We did not rinse or submerge the brain in liquid after removal.

### Splenocyte Isolation

The spleen was immediately rinsed in ice cold 1× PBS and transferred to a petri dish with ~ 5 mL DMEM (11320033, Thermo Fisher Scientific) supplemented with 2% FBS (76419-584, Avantor, Radnor Township, PA, USA). Connective tissue was removed, and spleens were cut into ~ 12 pieces. Each piece was gently dissociated between the rough ends of two frosted microscope slides. The resulting cell suspension was filtered through a pre-wet, 70 μm Nylon mesh filter. The suspension was centrifuged 300 × g for 3 minutes at RT. After discarding the supernatant, the cell pellets were gently resuspended, and 3 mL of RT 1× RBC Lysis buffer (00-4333-57, Thermo Fisher Scientific) was added for 45 seconds with gentle pipetting followed immediately by the addition of 10 mL cold PBS to quench the lysis. Tubes were centrifuged at 300 × g for 3 minutes at RT. The resulting pellets were resuspended in 2 mL PBS using a wide mouth 1 mL pipette tip, pooled, and filtered through a pre-wet, 70 μm nylon mesh. Cells were aliquoted into microcentrifuge tubes at 20 million cells per tube. Aliquots were centrifuged at 300 × g for 3 minutes at 4°C and the supernatant was discarded. Cell pellets were lysed for protein extraction or snap frozen and stored at −80°C until use.

### Western Blotting

The hippocampus and frontal cortex were dissected from the right hemisphere and lysed in 450 μL RIPA buffer (R0278-50ML, Sigma-Aldrich) with 50 μL phosphatase and protease inhibitors (08W00017, MP Biomedicals, Irvine, CA, USA) using a handheld pestle mixer. Samples were incubated on ice for 30 minutes, vortexed every 10 minutes before centrifuging at 14,000 × g for 15 minutes at 4°C. The supernatant was collected, aliquoted, and stored at −80°C until use. Four splenocyte pellets (80 million cells) per animal were lysed by pipetting cells up and down in 180 μL RIPA buffer with 20 μL phosphatase and protease inhibitors. Total protein concentrations were assessed using a Pierce BCA Protein Assay Kit (23227, Thermo Fisher Scientific). Western blot samples (brain: 22.5 μg for NLRP3 and IBA1 or 30 μg for IL-1β and caspase-1 per well, splenocytes: 15 μg per well) were prepared in 1× NuPage LDS Sample Buffer and 1× Reducing Agent, incubated at 95°C for 5 minutes loaded into wells of a NuPage Bis-Tris Midi Gel, 4–12% and run with MOPS running buffer. The Chameleon Duo Pre-Stained Protein Ladder (928-60000, LI-COR, Lincoln, NE, USA) was used and after electrophoresis, proteins were transferred from the gel to PVDF membranes with an iBlot Dry Blotting System (IB401001 & IB1001, Thermo Fisher Scientific) on Program 3 for 6 minutes (5.5 minutes for the second gel). Membranes were allowed to dry fully before continuing.

Total protein staining was used for normalization and visualized with Revert 700 Total Protein Stain (926-11021, LI-COR). Membranes were rinsed in TBS before blocking with 50% Intercept Blocking Buffer (927-60001, LI-COR) in TBS for 1 hour. Next, membranes were incubated with primary antibodies: mouse anti-NLRP3 (AG-20B-0014-C100, 1:1000 brain, 1:2000 spleen, Adipogene, San Diego, CA, USA), mouse anti-caspase-1 (AG-20B-0042-C100, 1:500, Adipogene), rabbit anti-IBA1 (17198T, 1:1000, Cell Signaling), and rabbit anti-IL-1β (ab283818, 1:1000 brain, 1:2000 spleen, Abcam) in the blocking buffer with 0.1% Tween-20 overnight at 4°C. Membranes were washed in TBS-T solution and incubated with the appropriate secondary antibodies - IRDye 800CW Goat anti-Mouse IgG and anti-Rabbit IgG and 680RD Goat anti-Mouse IgG and anti-Rabbit IgG (926-32210, 926-32211, 926-68070, and 926-68071, 1:10000, LI-COR) in the blocking buffer with 0.1% Tween-20 and 0.01% SDS for 1 hour at RT. They were then washed again in TBS-T before washing in TBS and then ddH_2_O for 5 minutes each and imaging. Blots were always left to incubate or wash on a shaker. The LI-COR Odyssey CLx (9140, LI-COR) was used to image the blots and ImageStudio Software (version 6.0.0.28) was used to quantify total protein and protein bands. Uncropped blots showing total protein staining and antibody signaling can be found in Supplementary Figs. 7–11.

### RNA Extraction

Immediately after brain removal, the hippocampus and frontal cortex were dissected from the left hemisphere and lysed in 1 mL of Trizol Reagent (15596026, Thermo Fisher Scientific) using a handheld pestle mixer, incubated for 5 minutes at RT, and snap frozen and stored at −80°C until use. RNA was extracted as per manufacturer’s instructions, with one microliter of GlycoBlue (AM9515, Thermo Fisher Scientific) added as a carrier. For splenocyte samples, RNA was extracted from frozen splenocyte pellets after thawing on ice for 10 minutes, using the RNeasy Plus Mini kit (04053228006138, QIAGEN, Hilden, Germany).

The RNA quality was assessed using the Nanodrop One (13-400-5181P5, Thermo Fisher Scientific). Samples achieving an OD260/280 greater than 2.0 and OD260/230 between 1.9 and 2.3 were deemed acceptable. Otherwise, samples were cleaned up with the Monarch RNA Cleanup Kit (T2050L, New England Biolabs, Ipswich, MA, USA) and re-assessed prior to use.

### RNA Sequencing Library Preparation

Library preparation was conducted by Novogene Co. (Beijing, China). In summary, initial quality control was conducted using a Nanodrop and Agilent 5400 Bioanalyzer to ensure sample concentration, integrity, and purity. Messenger RNA was isolated using poly-T oligo-attached magnetic beads. The purified mRNA was then fragmented and reverse-transcribed to synthesize first-strand cDNA using random hexamer primers followed by the second cDNA synthesis. The double-stranded cDNA underwent end-repair, A-tailing, and adapter ligation prior to size selection, PCR amplification, and purification. The library quality was checked with Qubit and a Bioanalyzer and quantified using real-time PCR. Quantified libraries were pooled and sequenced on an Illumina Novaseq X Plus to generate 150 base pair reads. Raw sequencing data are available on BioProject PRJNA1357591.

### RNA Sequencing Processing and Quality Control

Nextflow nf-core/rnaseq (v3.18.0-gb96a753)^118,119^ pipeline built with Nextflow (v24.10.5, build 5935) was used to process FASTQ files using default settings. Samples from each tissue type were processed separately. Briefly, Trim Galore! (v0.6.10) was used for adapter trimming, STAR (v2.7.11b) was used for alignment to *Rattus norvegicus* genome assembly GRCr8 (Ensembl 114)^120^, and Salmon (v1.10.3) was used to quantify transcripts. MultiQC^121-124^ reports indicated excessively elevated levels of read duplication for four samples in cortex and one sample in the hippocampus. Furthermore, one spleen sample displayed relatively short inner distance between two paired RNA reads, potentially indicative of degradation or biased library preparation. These six samples were excluded from further analyses. MultiQC reports including software versions and workflow summary are available on GitHub.

### RNA Sequencing Analysis

Low expression genes in each tissue were filtered out as previously described^125^. In specific, only transcripts with 10 or more counts in at least 50% of the samples in that tissue/subregion were included. DESeq2 (v1.28.0)^126,127^ was used to perform differential gene expression analysis between treatments in each tissue. DESeqDataSet objects were used for downstream analysis. Gene ranks derived from DESeq2 objects were used to conduct gene set enrichment analysis^128,129^ using the fgsea R package (v1.34.2)^130^. Tested gene sets include the Molecular Signatures Database (MSigDB)^131^ mouse collection: specifically, the Hallmark, Gene Ontology (GO), Reactome, and immunological signature gene sets. Only pathways with at least 5 and no more than 500 genes were included^132^. Additionally, a manual curation of rat brain gene sets (Brain.GMT) was tested with the brain tissue samples^77^. All reported p-values are the FDR-adjusted p-values from the packages.

### Statistical Analysis

Statistical analysis, excluding RNA-Seq, was performed using the statsmodels and scipy.stats packages in Python 3.12.4 or GraphPad Prism9 (version 9.5.1). Assumptions of normality were assessed using the Shapiro-Wilk test and homogeneity of variance was tested with Levene’s test or Spearman’s test for heteroscedasticity. A significance level of α = 0.05 was considered statistically significant. For analysis with both sexes, two-way ANOVAs were run to assess the effect of LPS and sex as well as VNS and sex. If assumptions of normality/homogeneity of variance were violated, a two-way Aligned Rank Transform ANOVA was used. Benjamini-Hochberg False Discovery Rate (FDR) procedure was used to correct for multiple comparisons. In the case of a significant interaction, Student’s t-tests were used to assess main effects for each sex. For western blot analyses with only males, one-way ANOVAs were used with Dunnett’s post-hoc test. All reported p-values in the manuscript and figures are after correction for multiple comparisons.

## Supplementary Material

This is a list of supplementary files associated with this preprint. Click to download.

- nrreportingsummaryfilledout.pdf
- SupplementaryData1.xlsx
- SupplementaryData2.xlsx
- SupplementaryData3.xlsx
- SupplementaryInformation.pdf

## Data Availability

Numerical data supporting the findings of this study are included in Supplementary Data 1–3. RNA-Seq data have been deposited in the National Center for Biotechnology Information Sequencing Read Archive (SRA) under BioProject PRJNA1357591. All other data are available from the corresponding authors upon reasonable request.

## Code Availability

Scripts for analysis and visualization of RNA-Seq data are available at http://github.com/irikas/2025_GL_RatSeq.
